# Clinical standards for the diagnosis, treatment and prevention of TB infection

**DOI:** 10.5588/ijtld.21.0753

**Published:** 2022-03-01

**Authors:** G. B. Migliori, S. J. Wu, A. Matteelli, D. Zenner, D. Goletti, S. Ahmedov, S. Al-Abri, D. M. Allen, M. E. Balcells, A. L. Garcia-Basteiro, E. Cambau, R. E. Chaisson, C. B. E. Chee, M. P. Dalcolmo, J. T. Denholm, C. Erkens, S. Esposito, P. Farnia, J. S. Friedland, S. Graham, Y. Hamada, A. D. Harries, A. W. Kay, A. Kritski, S. Manga, B. J. Marais, D. Menzies, D. Ng, L. Petrone, A. Rendon, D. R. Silva, H. S. Schaaf, A. Skrahina, G. Sotgiu, G. Thwaites, S. Tiberi, N. Tukvadze, J.-P. Zellweger, L. D’Ambrosio, R. Centis, C. W. M. Ong

**Affiliations:** 1 Respiratory Diseases Clinical Epidemiology Unit, Istituti Clinici Scientifici Maugeri IRCCS, Tradate, Italy; 2 Division of Infectious Diseases, Department of Medicine, National University Hospital, National University Health System, Singapore City; 3 Division of Infectious and Tropical Diseases, Spedali Civili University Hospital, Brescia, Italy; 4 WHO Collaborating Centre for TB/HIV Collaborative Activities and for TB Elimination Strategy, University of Brescia, Brescia, Italy; 5 Centre for Global Public Health, Institute for Population Health Sciences, Queen Mary University, London, UK; 6 Translational Research Unit, National Institute for Infectious Diseases “Lazzaro Spallanzani”, IRCCS, Rome, Italy; 7 USAID, Bureau for Global Health, TB Division, Washington, DC, USA; 8 Directorate General for Disease Surveillance and Control, Ministry of Health, Muscat, Oman; 9 Infectious Disease Translational Research Programme, Department of Medicine, National University of Singapore, Yong Loo Lin School of Medicine, Singapore City; 10 Department of Infectious Diseases, School of Medicine, Pontificia Universidad Católica de Chile, Santiago, Chile; 11 Centro de Investigação em Saúde de Manhiça (CISM), Maputo, Mozambique; 12 ISGlobal, Barcelona Centre for International Health Research, Hospital Clínic - Universitat de Barcelona, Barcelona, Spain; 13 IAME UMR1137, INSERM, University of Paris, F-75018 Paris; AP-HP-Bichat Hospital, Associate laboratory of National Reference Center for Mycobacteria and Antimycobacterial Resistance, Paris, France; 14 Center for Tuberculosis Research, Johns Hopkins University School of Medicine, Baltimore, MD, USA; 15 Tuberculosis Control Unit, Tan Tock Seng Hospital, Singapore, Singapore; 16 Helio Fraga Reference Center, Oswaldo Cruz Foundation Ministry of Health, Rio de Janeiro, Brazil; 17 Victorian Tuberculosis Program, Melbourne Health, Melbourne, VIC, Australia; 18 Department of Infectious Diseases, Peter Doherty Institute for Infection and Immunity, University of Melbourne, Melbourne, Australia; 19 KNCV Tuberculosis Foundation, The Hague, The Netherlands; 20 Paediatric Clinic, Pietro Barilla Children’s Hospital, University of Parma, Parma, Italy; 21 Mycobacteriology Research Center (MRC), National Research Institute of Tuberculosis and Lung Disease (NRITLD), Shahid Beheshti University of Medical Sciences, Tehran, Iran; 22 Institute for Infection and Immunity, St George’s, University of London, London, UK; 23 Department of Paediatrics, Center for International Child Health, University of Melbourne, Melbourne, VIC, Australia; 24 Murdoch Children’s Research Institute, Royal Children’s Hospital, Melbourne, Australia; 25 Institute for Global Health, University College London, London, UK; 26 International Union Against Tuberculosis and Lung Disease, Paris, France; 27 Department of Clinical Research, Faculty of Infectious and Tropical Diseases, London School of Hygiene & Tropical Medicine, London, UK; 28 The Global Tuberculosis Program, Texas Children’s Hospital, Department of Pediatrics, Baylor College of Medicine, Houston, TX, USA; 29 Academic Tuberculosis Program Center, Faculty of Medicine, Federal University of Rio de Janeiro, Rio de Janeiro, RJ, Brazil; 30 Operational Center, Medecins Sans Frontieres (MSF), Paris, France; 31 Department of Infectious Diseases and Microbiology, The Children’s Hospital at Westmead, Westmead, NSW, Australia; 32 The University of Sydney Institute for Infectious Diseases, Sydney, NSW, Australia; 33 Montréal Chest Institute, Montréal, QC, Canada; 34 Respiratory Epidemiology and Clinical Research Unit, Centre for Outcomes Research and Evaluation, Research Institute of McGill University Health Centre, Montréal, QC, Canada; 35 McGill International Tuberculosis Centre, Montréal, QC, Canada; 36 Infectious Diseases, National Centre for Infectious Diseases, Singapore; 37 Centro de Investigación, Prevención y Tratamiento de Infecciones Respiratorias CIPTIR, University Hospital of Monterrey UANL (Universidad Autonoma de Nuevo Leon), Monterrey, Mexico; 38 Faculdade de Medicina, Universidade Federal do Rio Grande do Sul (UFRGS), Porto Alegre, RS, Brazil; 39 Desmond Tutu TB Centre, Department of Paediatrics and Child Health, Faculty of Medicine and Health Sciences, Stellenbosch University, Cape Town, South Africa; 40 Republican Research and Practical Center for Pulmonology and Tuberculosis, Minsk, Belarus; 41 Clinical Epidemiology and Medical Statistics Unit, Department of Medical, Surgical and Experimental Sciences, University of Sassari, Sassari, Italy; 42 Oxford University Clinical Research Unit, Ho Chi Minh City, Vietnam; 43 Centre for Tropical Medicine and Global Health, Nuffield Department of Medicine, University of Oxford, Oxford, UK; 44 Department of Infection, Royal London Hospital, Barts Health NHS Trust, London, UK; 45 Blizard Institute, Queen Mary University of London, London, UK; 46 National Center for Tuberculosis and Lung Diseases, Tbilisi, Georgia; 47 TB Competence Center, Swiss Lung Association, Berne, Switzerland; 48 Public Health Consulting Group, Lugano, Switzerland; 49 National University of Singapore Institute for Health Innovation & Technology (iHealthtech), Singapore, Singapore

**Keywords:** tuberculosis, TB preventive therapy, TB infection tests, clinical standards

## Abstract

**BACKGROUND:**

Tuberculosis (TB) preventive therapy (TPT) decreases the risk of developing TB disease and its associated morbidity and mortality. The aim of these clinical standards is to guide the assessment, management of TB infection (TBI) and implementation of TPT.

**METHODS:**

A panel of global experts in the field of TB care was identified; 41 participated in a Delphi process. A 5-point Likert scale was used to score the initial standards. After rounds of revision, the document was approved with 100% agreement.

**RESULTS:**

Eight clinical standards were defined: Standard 1, all individuals belonging to at-risk groups for TB should undergo testing for TBI; Standard 2, all individual candidates for TPT (including caregivers of children) should undergo a counselling/health education session; Standard 3, testing for TBI: timing and test of choice should be optimised; Standard 4, TB disease should be excluded prior to initiation of TPT; Standard 5, all candidates for TPT should undergo a set of baseline examinations; Standard 6, all individuals initiating TPT should receive one of the recommended regimens; Standard 7, all individuals who have started TPT should be monitored; Standard 8, a TBI screening and testing register should be kept to inform the cascade of care.

**CONCLUSION:**

This is the first consensus-based set of Clinical Standards for TBI. This document guides clinicians, programme managers and public health officers in planning and implementing adequate measures to assess and manage TBI.

## Abstract (fr)

**CONTEXTE:**

L’objectif de ces normes cliniques est d’orienter l’évaluation et la prise en charge de l’infection tuberculeuse (TBI), ainsi que la mise en place du traitement préventif antituberculeux (TPT). Le TPT réduit le risque de TB active et de morbidité et mortalité associées.

**MÉTHODES:**

Un panel d’experts internationaux en matière de soins antituberculeux a été identifié ; 41 experts ont participé à un processus Delphi. Une échelle de Likert à 5 points a été utilisée pour noter les normes initiales. Après plusieurs révisions, le document a été approuvé par tous les experts (100%).

**RÉSULTATS:**

Huit normes cliniques ont été définies : Norme 1, tous les individus des groupes à risque de TB doivent subir un test de dépistage de la TBI ; Norme 2, tous les candidats au TPT doivent prendre part à une consultation de santé informative/éducative (y compris les aidants d’enfants) ; Norme 3, test de dépistage de la TBI : le moment et le test de référence choisi doivent être optimisés; Norme 4, une TB active doit être écartée avant d’instaurer un TPT ; Norme 5, tous les candidats au TPT doivent subir différents examens initiaux ; Norme 6, tous les individus démarrant un TPT doivent recevoir l’un des protocoles recommandés ; Norme 7, tous les individus ayant démarré un TPT doivent subir des examens de contrôle ; Norme 8, un dépistage de la TBI doit être réalisé et un registre des tests doit être tenu pour orienter la cascade de soins.

**CONCLUSION:**

Il s’agit du premier ensemble de normes cliniques pour la TBI fondées sur un consensus. Notre objectif est d’améliorer les soins et la qualité de vie des patients en orientant les cliniciens, responsables de programme et agents de santé publique en matière de planification et de mise en place de mesures adéquates d’évaluation et de prise en charge de la TBI.

In 2020, the World Health Organization (WHO) estimated that approximately 5.8 million individuals were diagnosed with TB disease, with 1.5 million deaths (including those co-infected with HIV). These numbers may be underreported, as the COVID-19 pandemic has led to an underdiagnosis of TB cases, which will likely lead to increased spread of TB infection (TBI) and disease.^1^ It is estimated that one quarter of the global population is infected with *Mycobacterium tuberculosis* (Mtb).^2^ Transmission of Mtb is predominantly through the respiratory route: bacilli generated by infectious individuals when coughing, speaking, singing, sneezing, and even breathing are acquired by contacts who inhale droplet nuclei of 1–5 μm.^3^,^4^ Mtb infection may be rapidly cleared by the innate immune response, or infection may occur leading to either a TB infection TBI, or to TB disease.^5^,^6^

An estimated 20–25% of individuals become infected following exposure to Mtb. Among these individuals, the risk of TB disease within the first 5 years is 5–15%.^7^ The innate and adaptive immune systems of an infected person control the replication of the pathogen.^8^ Different risk/predisposing factors can play a role in Mtb progression, including coinfection with HIV or other pathogens (e.g., measles and possibly SARS-CoV-2), young age, comorbidities (e.g., diabetes mellitus [DM], renal insufficiency and malignancies), genetic factors (e.g., primary immunodeficiencies), malnutrition, immunosuppression with biological agents (e.g., anti-tumour necrosis factor-α agents), harmful habits (e.g., smoking, substance abuse, harmful use of alcohol) and solid organ and haematological transplantation.^5^,^9^–^12^ The management of TBI is described under Pillar One of the WHO’s End TB Strategy (Integrated, patient-centred care and prevention) and is a core intervention in pursuit of TB elimination.^13^–^15^ Our aim is to define the clinical and public health standards for TBI management.

Standards are different from guidelines, and instead present a widely accepted level of diagnosis and care, for *all* healthcare providers and clinicians, both public and private, for appropriate management in patients who have, or who are presumed to suffer from, a given disease (or infection).^16^–^19^ The IJTLD Clinical Standards complement existing WHO or other guidelines and integrate their recommendations to provide a specific clinical focus. The standards are universal principles and might need to be adapted to specific settings and situations for future programmatic implementation due to legal, organisational or economic reasons.

Specific evidence in some areas is still limited (e.g., which diagnostic test to perform before starting TPT, or during TBI treatment monitoring). The clinical standards are based on best available evidence and will be updated to capture new evidence as it accumulates over time.

## AIM OF THE CLINICAL STANDARDS

This consensus-based document describes the following activities:

1. Identifying the population groups that need to be tested (Standard 1). A universal standard was defined, with special considerations for children and possible adaptation in different settings and situations (for organisational, legal or economic reasons).
2. Proposing education and counselling for individuals and caregivers of children undergoing TPT (Standard 2).
3. Defining when TBI testing should be done and specifying which test(s) to perform (Standard 3).
4. Excluding TB disease prior to the initiation of TPT (Standard 4).
5. Defining the set of baseline examinations (and tests) that individuals initiating TPT should undergo (Standard 5).
6. Choosing the appropriate TPT regimen (Standard 6).
7. Defining the set of monitoring examinations that individuals taking TPT should undergo (Standard 7).
8. Defining public health priorities (cascade of care and implementation of the TBI register) (Standard 8).

Priorities for future research in TBI are also discussed.

## METHODS

A panel of global experts was identified to represent the main scientific societies, associations and groups active in the field of TB. Of the 62 experts initially invited, 51 agreed to participate and 11 did not respond after one reminder. The 51 respondents were asked to comment via a Delphi process on an initial draft of 11 standards developed by a core coordination team (composed of eight members). Forty-one experts provided valid answers after one reminder. The final panel included TB clinicians (*n* = 17), TB public health specialists (*n* = 15), TB paediatricians (*n* = 4), microbiologists/biologists (*n* = 3) and methodologists (*n* = 2). A 5-point Likert scale was used (5: high agreement; 1: low agreement). At the first Delphi round, agreement was high, with a median value of 4–5 for all standards, except for the public health standard (Standard 8). The agreement (defined as score ≥3) was over 95% for all standards and 73% for the public health standard. Based on substantial agreement on the Standards and on the document outline, a draft document was jointly developed by the expert panel, reducing the original 11 standards to eight. The document underwent nine rounds of revision and the final version was approved by consensus (100% agreement).

## STANDARD 1

### All individuals belonging to at-risk groups for TB should undergo testing for TBI

The WHO outlined the risk/predisposing factors, and the following population groups are at high risk for progression to TB disease or high rates of TBI (Table 1):^20^

**Table 1:** Groups at risk for TB disease and recommended for TBI testing

Groups at risk for TBI and disease due to close contact with infectious TB patients
Adult and child (especially <5 years of age) TB contacts of known infectious TB patients
Healthcare workers and students
Migrants from countries with a high TB burden (TB incidence >100/100,000 population)
Homeless people
Prisoners
Groups at high risk for TB development after *M. tuberculosis* infection: immunocompromised hosts
People living with HIV
Patients on renal dialysis
Patients initiating therapy with TNF-α inhibitors
Patients preparing for organ or haematological transplant
Patients with silicosis
People who abuse drugs
People with DM, engage in the harmful use of alcohol, tobacco smokers and severely malnourished belonging to the above categories

TBI = TB infection; TNF = tumour necrosis factor; DM = diabetes mellitus.

1. Adults, adolescents, and children living with HIV who are unlikely to have TB disease on clinical evaluation;
2. Household or close contacts of people with bacteriologically confirmed pulmonary TB (all ages, but especially young children <5 years of age);
3. People who are initiating treatment with tumour necrosis factor-α (TNF-α) inhibitors, receiving dialysis, preparing for an organ or haematological transplant, or who have silicosis;
4. Prisoners, healthcare workers who have frequent unprotected contact with TB patients, migrants from countries with high TB burden, homeless people and people who abuse drugs.

People with DM, those who engage in the harmful use of alcohol, tobacco smokers and underweight people have a higher risk for TB progression than the general population; however, systematic TBI testing and treatment is not recommended unless they also belong to other risk groups described above (Table 1).^11^,^20^,^21^

Infection and subsequent reactivation of TBI is a probabilistic phenomenon, involving many different factors.^22^–^24^ An individual with respiratory TB disease generates infectious droplet nuclei, the number of which depends on the cough strength, presence of lung cavities, sputum viscosity and ventilation.^3^,^9^

The contact needs to inhale infectious droplet nuclei, each containing at least one inhaled viable and virulent Mtb bacillus (via one or more exposures), which must reach the alveolar macrophages in the lung to become infected. To interrupt this chain of exposure, it is crucial that the source patient receives prompt and effective TB treatment and that appropriate infection control measures are in place. Rigorous monitoring of the different steps in the cascade of care is further elaborated in Standard 8.

## STANDARD 2

### All individual candidates for TPT (including caregivers of children) should undergo a counselling/health education session

Individuals who undergo TBI testing should receive TPT if tested positive (i.e., a decision to test should be a decision to treat). Each individual considered for TPT should receive counselling and health education, including a specific session during the follow-up for treatment monitoring, organised according to feasibility and cost-effectiveness criteria, based on the availability of health services and tailored to the individual’s needs. Health education is essential for TPT adherence and in ensuring safety; not only does it provide instructions for reporting adverse events but also informs eligible persons on individual risks and benefits, thereby motivating treatment completion. The ease of contact with a healthcare provider would enable the individual to report any adverse effects from drugs used for TPT.^25^ Components of education include information on TB, its mode of transmission, prevention, and drugs available to treat both TBI and disease, and on when to contact the healthcare provider in the event of symptoms suggestive of adverse effects, or treatment interruptions. To adequately inform the individual and obtain the individual’s consent to TBI management (see also Standard 8), the difference between TB disease and TBI should be explained, highlighting that effective TPT reduces the risk of progression from TBI to TB disease. To facilitate early recognition of drug adverse effects and reporting to healthcare providers, major adverse effects of the TPT drugs should be explained.

Furthermore, the counselling and education session may be an opportunity to promote other healthy lifestyle behaviours such as good nutrition or smoking cessation, to discuss how to combine treatment intake with daily activities to improve treatment adherence and to highlight or better manage risk factors predisposing to TB disease (e.g., HIV, DM). Health education should also be provided to the exposed families and household members.^26^ This is especially important for children, as parents and/or other caregivers will be responsible for administering medication. Health education should be age-specific, gender-sensitive, delivered in the patient’s own language and address potential challenges for the caregiver when administering medications.^16^,^17^ The availability of palatable paediatric formulations and tolerability of medications would help adherence to treatment. Recommendations for an effective health education and counselling session are given in Table 2.

**Table 2:** Components of the health education and counselling session

Health education and counselling are highly recommended for successful implementation of TPT Health education is important to motivate the target population to be tested and participate in the programme Counselling subsequently occurs where those eligible for TPT are identified Key points for health education: Structured and comprehensive educational programmes are an integral and essential component of the management of TPT Educational programmes should be age-specific, gender and culturally sensitive, delivered in the local language and extended to mothers and families/households Education should be delivered by professionals who are competent in the relevant subject areas and trained to deliver educational sessions Educational materials and technological support used to deliver them needs to be evaluated in the setting-specific context Recommended topics for counselling: Basic principles of TB: epidemiology, clinical aspects and transmission routes Difference between TB disease and TB infection, role of TPT in reducing progression Importance of TPT (and treatment adherence/retention in care) to reduce the risk of developing TB disease in the presence of TBI Simple concepts of infection control and safety procedures Advantages/importance of smoking cessation and risk of comorbidities (e.g., HIV co-infection and DM) in household/families Ensuring adequate nutrition and refraining from alcohol consumption Importance of adhering to medical prescriptions for the management of comorbidities and vaccinations Recognition of drug adverse effects and the need to report to healthcare providers Information on how to contact the healthcare provider if needed Discussing with the individual what potential barriers are for TPT completion and how these can be addressed/overcome (adherence plan)

TPT = TB preventive therapy; TBI = TB infection; DM = diabetes mellitus.

## STANDARD 3

### Testing for TBI: timing and the test of choice should be optimised

Standard 1 outlined the population groups that should be carefully evaluated for TBI testing as it identifies individuals who are most likely to benefit from treatment.^20^ To date, the skin-based tuberculin skin test (TST) and blood-based interferon γ -release assays (IGRAs) are the two test categories widely used as a proxy for TBI diagnosis. These tests identify people who were infected with Mtb in the past or at present by measuring the immune response to the bacilli (Table 3). However, there is a 6–8-week conversion window for these tests; therefore, a reliable assessment cannot be made within 2 months of the last infectious exposure. Both TST and IGRAs identify individuals who are at risk of progression to TB disease and are equally recommended for TBI testing.^27^ Advantages and disadvantages are outlined in Table 3. The use of IGRAs is recommended for individuals who have been vaccinated with bacilli Calmette-Guérin (BCG) and would therefore cross-react to tuberculin; for those aged ≥5 years; and in settings where a cold chain cannot be maintained, as reagents for TST are temperature-sensitive.^9^,^20^,^28^ Limitations of TST include the need for two consecutive healthcare visits, the requirement for skilled personnel for TST application and analysis of the result at 48–72 h, and the recommended use of different cut-off points for TST positivity in various target populations, which makes interpretation more complex. However, IGRAs require phlebotomy, which requires a relatively large volume of blood from children, advanced laboratory equipment and technical expertise. Although more expensive than the TST, IGRAs require just one healthcare visit instead of two visits at specified time points, and are not observer-dependent, as these provide an objective result based on an internal control.^9^,^20^

**Table 3:** Tests for TB infection, advantages and disadvantages, and NPV and PPV (modified from Migliori et al. and Zhou et al.)^9^,^66^

Tests of infection	TST	IGRAs and in vitro tests (e.g., QuantiFERON-TB Gold Plus and T-SPOT.*TB*, Standard E TB-Feron, AdvanSure TB-IGRA ELISA, LIOFeron TB/LTBI, IP-10 ELISA)
Characteristics	Intradermal administration of PPD in the forearm Cold chain requirement for PPD Induration reading within 48–72 h Trained staff for administration and reading the skin induration	In vitro tests with internal and external controls Detection of IFN-γ or other markers (i.e., IP-10) in blood or PBMCs using ELISA or ELISPOT Need for fresh samples Incubation time of 16–24 h Need for efficient sample transport system Need for different blood collection tubes, depending on the altitude Need for laboratory infrastructure Assay time (after incubation): 20 min for IP-10 ELISA; 1.5–4 h for QuantiFERON-TB Gold Plus, T-SPOT.*TB*, Standard E TB-Feron, AdvanSure TB-IGRA ELISA, LIOFeron TB/LTBI For patients with a positive result, a second visit may also be needed to rule out TB disease and decide on further management
Disadvantages	False-positive results in BCG-vaccinated individuals and after exposure to non-tuberculous mycobacteria False-negative results in the immunocompromised hosts Inter-reader and intra-reader variability Second visit needed in a specific time period	False-negative results in the immunocompromised hosts Errors in the pre-analytical phase Expensive Phlebotomy Relatively large volume of blood in young children
Advantages	Useful for large screening settings Cost-effective No laboratory infrastructure required	Higher specificity for TB infection compared to TST No booster effect unlike TST No need of a second visit within specified time unlike TST
PPV for predicting development of TB disease^66^	2.3% (95% CI 1.5–3.1) TST induration 5 mm: 1.8% (95% CI 1.0–2.9) TST induration 10 mm: 2.9% (95% CI 2.1–3.7)	4.5% (95% CI 3.3–5.8) QFT-TB: 4.8% (95% CI 3.3–6.7) T-SPOT.*TB*: 3.9% (95% CI 2.7–5.4)
NPV for predicting development of TB disease^66^	99.3% (95% CI 99.0–99.5) TST 5 mm: 99.4% (95% CI 98.7–99.8) TST 10 mm: 99.2% 95% CI 98.9–99.4)	99.7% (95% CI 99.5–99.8) QFT-TB: 99.6% (95% CI 99.4–99.8) T-SPOT.*TB*: 99.8% (95% CI 99.6–100)

NPV = negative-predictive value; PPV = positive predictive value; TST = tuberculin skin test; IGRA = interferon-γ -release assays; ELISA = enzyme-linked immunosorbent assay; IP-10 = IFN-γ inducible protein; PPD = purified protein derivative; IFN-γ = interferon-gamma; PBMC = peripheral blood mononuclear cell; BCG = bacille Calmette-Guèrin; CI = confidence interval.

Newer and cheaper tests for TBI testing are needed to overcome the issues described. Newer skin tests and simplified IGRAs have been developed, but data on their performance are currently limited.^9^,^27^,^29^,^30^ There is provisional evidence on the performance of a lateral flow assay, which is a potential point-of-care test (compared to the commercially available IGRAs for both immunocompetent and immunocompromised patients), but it has not yet been endorsed by the WHO.^31^–^33^

## STANDARD 4

### TB disease should be excluded prior to the initiation of preventive therapy

**Figure 1:**
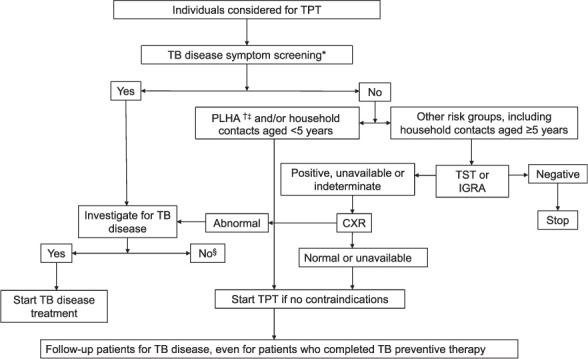
Algorithm to exclude TB disease before initiating TPT. *For adults and adolescents, including PLHA, a four-symptom screening of cough, fever, weight loss or night sweats is recommended. For infants and children living with HIV/AIDS and/or in contact with a person with infectious TB disease, screening for poor weight gain/loss of weight, fever, cough, decreased playfulness, symptoms of extrapulmonary TB (e.g., mass in neck, lethargy/sleepiness, vomiting) is recommended. ^†^CXR should be performed but lack of CXR should not be a barrier to initiating TPT in resource-limited settings. ^‡^TST or IGRA if available is ideal to identify PLHA who might benefit most from TPT. ^§^Patients should also be investigated for other diseases such as malignancies which have similar constitutional symptoms with TB disease. If clinical suspicion for TB disease is high but investigations are negative, a specialist may be consulted to determine if the patient should be empirically started on anti-TB treatment. In the event that TB disease is excluded, PLHA and household contacts ≤5 years old should be considered for TST/IGRA screening and TPT. TPT = TB preventive therapy; PLHA = patients living HIV/AIDS; TST = tuberculin skin test; IGRA = interferon-γ release assay; CXR = chest X-ray.

Existing TPT regimens utilise mono or dual-drug therapy. As a result, it is critical to ensure that TB disease is absent before commencing TPT to avoid undertreating TB disease and to eliminate the possibility of developing drug resistance (which would require treatment with a minimum of three, and preferably four effective WHO-recommended TB drugs).^34^ Screening for the four symptoms of cough, fever, weight loss and/or night sweats is the first key step in adults and adolescents, including patients living with HIV/AIDS (PLHA) regardless of antiretroviral treatment (ART) status.^20^,^35^,^36^ For infants and children living with HIV/AIDS and/or in contact with a person with infectious TB disease, screening for poor weight gain (failure to thrive) or weight loss, reduced playfulness or lethargy, fever and active cough is recommended; this is feasible in a contact-tracing scenario.^20^,^37^,^38^ Symptom screening and a chest X-ray (CXR) are strongly recommended before commencing TPT. Physical examination should also be conducted to ensure that there are no palpable lymphadenopathy suggestive of TB lymphadenitis or other signs of extrapulmonary TB disease. An evidence-based algorithm to exclude TB disease is shown in Figure 1.

We acknowledge that CXR and the expertise to interpret the CXR may not be readily available, especially in resource-limited settings. In such scenarios, the lack of CXR should not be a barrier to TPT, and symptom screening should be prioritised.^20^ Individuals with symptoms suggestive of TB disease should be investigated according to national guidelines, including PLHA with normal CXR, as it is well-recognised that TB disease may still be present.^39^ Constitutional symptoms may overlap with other diseases such as malignancies, which may also co-exist with TB disease; further investigations are therefore needed when TB investigations are negative.^40^ Those in whom TB disease and other differential diagnoses are excluded should be assessed for eligibility for TPT. The investigations that are used to exclude TB disease are shown in Table 4. Both CXR and chest computed tomography (CT) scans are well-established clinical tools to investigate TB disease. Computer-assisted diagnostic software such as the computer-aided detection of TB-related abnormalities on CXR (CAD) combines digital CXR with an algorithmic interpretation to provide a TB risk score; in some cases, this may be complemented by remote expertise in order to diagnose pulmonary TB promptly and select patients who should undergo further TB disease investigations. CAD has now been implemented in resource-limited settings, where automated interpretation and remote radiology may be particularly helpful.^41^ However, this technology is not validated for use in children nor for other pulmonary diseases that mimic TB.

**Table 4:** Investigations to exclude TB disease ^*^

Tests	Evidence	Limitations
CXR	Strongly recommended to exclude pulmonary TB disease. However, lack of availability should not be a barrier to TB preventive therapy	HIV-positive patients, especially those with low CD4 counts may have active pulmonary TB disease despite normal CXR Cost and lack of expertise to interpret may be a limitation in resource-limited settings
CT	Useful for investigating some types of extrapulmonary TB In the presence of constitutional symptoms and negative TB investigations, this can be useful to investigate for malignancies In high-income, low TB burden countries, may help to exclude pulmonary TB in children	Expensive, requires expertise to interpret Not readily available in resource-limited settings Risk of radiation
Computer-assisted diagnostic software e.g., CAD	Computer software that detects TB more accurately and cost-effectively. Combines digital X-rays with deep learning and remote expertise to diagnose pulmonary TB disease	Expensive Not validated in children
CRP	Conditional recommendation by WHO of cut-off >5 mg/dl	Specificity of CRP for TB screening among patients living with HIV was found to be extremely low, likely due to competing comorbidities that would also result in raised CRP levels and the presence of symptoms^20^ Not helpful in children as CRP can be normal or abnormal with TB disease

^*^ If physical examination reveals palpable lymphadenopathy, fine-needle aspiration cytology or biopsies can be performed to investigate for TB disease. CXR = chest X-ray; CT =computed tomography; CAD = computer-aided detection (of TB-related abnormalities on CXR); CRP = C-reactive protein.

#### Considerations for implementation

Pregnancy is not an absolute contra-indication to CXR. Additional measures, including a lead shield, may be used to protect the foetus with the CXR taken at full-inspiration. In asymptomatic at-risk individuals who test negative for TBI in the first instance, the frequency of symptom screening, TBI testing (TST or IGRA testing) and CXR are dependent on individual risk and would need to be defined by local health authorities. If CXR and/or IGRA/TST testing are unavailable in an asymptomatic individual who is at risk of progressing to TB disease, the WHO recommends that TPT should still be started.^20^ A history of treated TB disease or treated TBI are not contraindications to TPT and should be recommended in young children (<5 years of age), PLHA or individuals with silicosis, who may be at high risk of progression to TB disease if re-exposed to infectious TB patients.^42^,^43^

## STANDARD 5

### All candidates for TPT should undergo a set of baseline examinations

TPT regimens containing rifamycins and/or isoniazid (H, INH) may confer a small risk of hepatoxicity, which is age-related for INH.^44^ There is currently no strong evidence on the need for liver function tests at baseline, although this is recommended in many guidelines and protocols.^45^ However, individuals in the following categories should be closely monitored for drug-induced liver injury from TPT and undergo baseline liver function tests: history of liver disease, including viral hepatitis; harmful alcohol intake; age >35 years; pregnancy and up to 3 months postpartum; immunocompromised individuals, including PLHA; exposure to statin therapy; and the mentally incapacitated, who are unable to reliably report symptoms of hepatotoxicity (e.g., dementia) (Table 5). TPT with rifampicin (R, RIF) and/or INH can be started if liver enzymes are <3x upper limit of normal. In the context of drug resistance and a TPT with fluoroquinolones in adults, a baseline electrocardiogram (ECG) should be performed to ensure that the QTc interval is not prolonged (relative contraindication). Candidates should be counselled about the risks of tendinopathies in adults, while those with DM should also be informed on the risks of hypoglycaemia which may occur with use of fluoroquinolones.

**Table 5:** Baseline tests prior initiation of TB preventive treatment

Drug for TB preventive treatment	Baseline tests
Rifamycins (rifampicin, rifapentine)	AST and ALT for patients with: History of liver disease
Isoniazid	Harmful alcohol intake Age >35 years Pregnancy and in the immediate 3 months post-partum Immunocompromised individuals, including PLHA Receiving statin therapy Patients who are mentally incapacitated and unable to report symptoms of hepatotoxicity (e.g., dementia)
Levofloxacin/moxifloxacin*	ECG for prolonged QTc interval^†^

* Hypoglycaemia is a recognised adverse effect of fluoroquinolones. Individuals should be advised to monitor their capillary blood, and to observe for symptoms suggestive of hypoglycaemia such as cold sweats, dizziness, tremors, palpitations, confusion and feeling hungry. In adults, tendinopathies may occur, and individuals should also be counselled.

^†^Prolonged QTc interval is a relative contraindication to fluoroquinolones. If this is present, alternative regimens should be considered, or patients should be monitored regularly with ECG for arrhythmias. ECG is not required for children.

AST = aspartate aminotransferase; ALT = alanine aminotransferase; PLHA = people living with HIV and AIDS; ECG = electrocardiogram.

#### Considerations for implementation

We recognise that baseline liver function tests, and ECG may not be routinely available in all settings. This should not be a reason to withhold TPT. Counselling individuals should be done regardless of baseline tests, symptoms suggestive of drug-induced liver injury (e.g., new onset nausea and/or vomiting, abdominal pain, jaundice, tea-coloured urine, which should be differentiated from the expected orange-coloured urine if receiving a rifamycin), or palpitations suggestive of arrhythmias if receiving fluoroquinolones. Should abnormalities be found, patients are to be regularly monitored throughout TPT, as described in Standard 7. All patients should be informed of potential serious adverse effects and be instructed to immediately stop TPT and communicate with their healthcare provider should potentially severe adverse effects occur.

## STANDARD 6

### Individuals initiating TPT should receive one of the following regimens

The following options are recommended by the WHO for the treatment of drug-susceptible TBI.^20^

- 3 months of daily INH plus RIF (3HR)
- 4 months of daily RIF alone (4R)
- 6 months of daily INH (6H)
- 12 weeks of once-weekly rifapentine (P, RPT) plus INH (3HP)
- 1 month of daily RPT plus INH (1HP)

A regimen of 36 months of daily INH may be given to adults and adolescents living with HIV in settings with very high risk of TB transmission. Also, in selected high-risk household contacts of patients with multidrug-resistant TB (MDR-TB), the following TPT may be considered based on an individualised risk assessment and a sound clinical justification (especially in children less than 5 years of age): 6 months of daily levofloxacin with or without ethambutol or another drug to which the source case’s Mtb isolate is susceptible.

TPT for an infection with strains presumed to be drug-susceptible can be broadly categorised into two types: 1) treatment with regimens containing a rifamycin (RIF or RPT) of 1–4 months’ duration, or 2) monotherapy with INH for at least 6 months. A summary of the regimen and the evidence for each regimen is presented in Table 6.

**Table 6:** Treatment regimens for TB infection

Regimen	Dosing and duration	Remarks
Rifamycin based regimens (potent cytochrome P-450 inducers)		
RIF + INH (3RH)	3 months For adults: RIF 10 mg/kg (max dose 600 mg) OD; INH 5 mg/kg (max dose 300 mg) OD For children: RIF 10–20 mg/kg (max dose 600 mg) OD; INH 7–15 mg/kg (max dose 300 mg) OD	Equivalent to 6H in efficacy and safety^67^ Higher adherence and better tolerability in the paediatric population^68^
RIF monotherapy (4R)	4 months RIF 10 mg/kg (max dose 600 mg) OD	Non-inferior to 9H^69^ Better tolerability and higher completion rates^70^
RPT + INH (weekly) (3HP)	3 months RPT ≤32 kg: 600 mg once/week 32.1–49.9 kg: 750 mg once/week ≥50 kg: 900 mg once/week INH 15 mg/kg (max dose 900mg) once/week Not recommended for children <2 years (studies ongoing)	When compared to 6H/9H, 3HP showed significantly lower risk of hepatotoxicity and had a higher completion rate,^71^–^74^ with no significant difference in developing TB disease Decreased pill burden RPT is currently not readily available worldwide and is costly
RPT + INH (daily) (1HP)	1 month RPT <35 kg: 300 mg OD 35–45 kg: 450 mg OD >45 kg: 600 mg OD INH 300 mg OD Not recommended for children <2 years (studies ongoing)	Shortest option available and non-inferior to 9H in PLHA with similar treatment completion rates. A prospective cohort study on children aged 2–19 years showed that the regimen is safe^47^,^75^
Non-rifamycin-based regimens		
INH monotherapy (6H)	6 months INH 5 mg/kg (max dose 300 mg) OD, OR INH 15 mg/kg (max dose 900 mg) twice/week Children: 7–15 mg/kg	Mainstay of TB preventive therapy with a systematic review showing reduction in TB disease incidence in those given 6H compared to placebo^67^,^76^ Significant risk of liver toxicity, that may be fatal. 9 months regimen based on re-analysis of trials from 1950s and 1960s showed a progressive benefit of INH when given up to 9–10 months;^77^ however, in the absence of clinical trials comparing 6H to 9H, 6H is preferred Regimen is also preferred if there are concomitant drugs that interact with rifamycins
INH monotherapy (preventive regimen) (IPT)	36 months	Used as a preventive strategy in specific areas and during periods of intense TB transmission Meta-analysis of 3 RCTs of PLHA in settings with high TB prevalence, 36 months IPT reduced risk of TB disease by 38% more than 6H^78^ Continuous INH monotherapy may reduce rate of TB infection or death^73^
MDR-TB		
FQ with/without a second drug to which the source case’s isolate is susceptible	Duration to be determined (6–12 months)	No RCT available; however, a prospective cohort study showed that no close contacts who received FQ developed TB disease vs. 20% who did not receive FQ developed TB;^79^ other RCTs on levofloxacin as a single drug to prevent MDR-TB are awaited Reduction in confirmed or probable TB in children has been shown^80^

RIF = rifampicin; INH = isoniazid; OD = once daily; RPT =rifapentine; PLHA = people living with HIV/AIDS; IPT = isoniazid preventive therapy; RCT =randomised-controlled trial; MDR-TB = multidrug-resistant TB; FQ = fluoroquinolone.

#### Considerations for implementation

Benefits outweigh potential harms for all recommended regimens. Regimens should be chosen considering the individual’s characteristics such as age, risk of toxicity or drug-drug interaction, comorbidities, drug susceptibility of the likely source strain, drug availability and availability of appropriate formulations (i.e., fixed-dose combination [FDC] pills). The choice is also determined by programmatic factors such as the choice to use or not to use rifamycin-containing regimens for prevention. The WHO recommends that national TB programmes should progressively transition to shorter rifamycinbased regimens given the better safety profile and better prospects of TPT completion.^20^ 3HR is a convenient and safe regimen with a FDC pill(s) and higher rate of completion compared to 6H. 4R is likely to be better tolerated and safer than 3HR or 6H. However, there is concern regarding the use of RIF as a single drug in high-prevalence, low-resource countries, where exclusion of TB disease may be problematic, or in settings where RIF is readily available as an over-the-counter drug without the need for prescriptions from a medical practitioner. In such settings, there may be an increase in RIF -resistant TB. The 6H (or 9H) regimen has long been the only available regimen, and is therefore well studied. The reasons to choose this regimen presently are mainly due to fewer drug–drug interactions than rifamycin regimens (especially in PLHA on protease inhibitor-based regimen, nevirapine, or integrase inhibitors due to potential drug–drug interactions), scarcity of alternative regimens, mainly in low-resource countries. The WHO had noted that all regimens can be self-administered, and that directly -observed-therapy (DOT) would pose a significant barrier to implementation.^20^ Individuals should be supported through education and counselling as described in Standard 2.

Newer studies show that RPT-based regimens have better benefit/risk profile than INH monotherapy but its use is restricted by availability. 1HP, the shortest regimen, would offer significant advantages. However, its effectiveness, especially in different populations at risk (e.g., young children), remains to be confirmed by future studies. Its cost and limited availability are a barrier for resource-limited settings. In general, for migrants, the homeless and in current or former prisoners in whom there is limited opportunity for follow-up during treatment, a shorter treatment may be more suitable. Although a systematic review conducted in 2019 did not confirm higher risk for severe adverse effects in pregnant women taking INH, there were two deaths in the INH arm in a large randomised controlled trial (RCT) in HIV pregnant women.^46^ INH use in pregnancy may be associated with higher risks of liver failure, and close monitoring is therefore required.

While obtaining baseline liver function tests when INH preventive therapy in pregnancy is strongly encouraged, unavailability of liver function tests should not be a barrier, unless there are other risk factors for liver toxicity. RIF is considered safe in pregnancy. There is limited data on the pharmacokinetics and safety of RPT in pregnancy; therefore, the use of 1HP and 3HP in pregnancy should be avoided until there is more evidence on dosing and safety. Individuals at risk for peripheral neuropathy, such as those with malnutrition, those who engage in harmful use of alcohol, PLHA, those with renal failure or DM, and those pregnant or breastfeeding should receive pyridoxine (vitamin B6) when taking INH-containing regimens. Unavailability of pyridoxine should not be a reason to withhold TPT.

Regimens based on INH and RIF can be used in individuals of all ages. There are no or very limited data on the efficacy and safety of RPT in children aged <2 years, and both the 1HP and 3HP regimens are only recommended for use in children aged ≥2 years.^47^ In many countries, RPT and child-friendly formulations are not available. Continuous drug supply needs to be guaranteed, as well as an adequate budget for training, dedicated staff and other programmatic needs.

FDCs of 3HR, in doses appropriate for children and adults, should be used where possible to reduce the number of tablets to be taken. FDCs of 3HP are expected to be available in the near future, which will facilitate administration. There is no evidence that TPT increases the risk of development of drug resistance, provided that TB disease has been excluded by symptom screening and CXR before the initiation of therapy (inadvertent monotherapy).^48^–^50^ The WHO has not recommended repeated courses of preventive therapy due to limited evidence.^20^

## STANDARD 7

### Individuals who have started TPT should be monitored

#### Monitoring toxicity

Most individuals who receive TPT are healthy, and adverse effects may influence their likelihood of completing it. Risks of drug-related toxicity should therefore be minimised by informing individuals receiving TPT to avoid alcohol and other drugs that interact with TPT. Individuals on TPT should ideally be monitored clinically at regular monthly intervals, either by phone or text messaging or other modalities according to feasibility. For individuals with abnormal baseline test results, sound clinical judgement is required to ensure that the benefit of TPT outweighs the risks, and they should be tested routinely. Access to informed healthcare providers and appropriate laboratory testing should also be performed for patients who become symptomatic while on treatment (e.g., liver function tests for those with symptoms of hepatotoxicity such as nausea, vomiting and jaundice/tea-coloured urine). Adverse effects should be closely followed. For example, it is recommended to stop a rifamycin and/or INH-containing regimen when there is an increase in transaminases to 5 times the upper limit of normal or to 3 times with symptoms of hepatotoxicity.

TPT regimens are generally safe and well-tolerated, but adverse effects, including hypersensitivity reactions, have been associated with INH (asymptomatic elevation of serum liver enzymes up to 5 times the upper limit of normal, peripheral neuropathy and hepatotoxicity) and RIF and RPT (flu-like symptoms, cutaneous reactions, hypersensitivity reactions, gastrointestinal intolerance and hepatotoxicity). Although most of these reactions are minor and occur rarely, specific attention should be paid to hepatic dysfunction to prevent drug-induced liver injury (Table 5). If liver function tests are unavailable and subjects were to develop nausea, vomiting, jaundice or confusion indicative of hepatitis or acute liver failure, then TPT should be ceased and such persons referred for further assessment immediately. Adverse effects should be monitored according to the WHO framework for monitoring and managing the safety of medicines against TB disease.^51^

#### Monitoring for TB disease

All patients receiving TPT should be clinically observed for TB disease while on TPT (history of symptoms, clinical signs), and further investigations done as clinically indicated. In people diagnosed with MDR-TB, strict clinical observation and close monitoring for TB disease based on sound clinical practice and national guidelines is required for at least 2 years after MDR-TB exposure, regardless of whether or not preventive treatment was given. More evidence for the effectiveness and safety of MDR-TB preventive treatment is urgently needed. The issue of the type of TPT for pre-extensively drug-resistant TB (pre-XDR-TB; i.e., fluoroquinolone-resistant MDR-TB) and XDR-TB (i.e., fluoroquinolone plus either bedaquiline- or linezolid-resistant MDR-TB) will be addressed once data are available.

#### Monitoring adherence

Regular follow-up, ideally at monthly intervals, is required to support and increase adherence; this can be based on digital technology/telehealth. Monitoring adherence to TPT and ensuring its completion will benefit the individual receiving it. A method to record data on the occurrence and management of adverse effects is advised. There is no data on how to handle interruptions to TPT, i.e., how many missed doses can be replaced by prolonging treatment without compromising efficacy.^20^ The WHO provides guidance on the management of treatment interruptions and defines treatment completion as 80% of recommended doses in a maximum number of days, which can be used as a guide.^20^ Data for management of non-adherence to the shorter 1HP or 3HP regimes are also needed. Given the absence of a reliable test that can ascertain infection clearance, maximising adherence is especially important. Interventions to enhance adherence and completion of treatment should be tailored to the specific needs of risk groups, including migrants, be culturally appropriate and in the language of the individual. A systematic review conducted for the WHO 2015 TBI guidelines provided heterogeneous results for interventions to improve treatment adherence and completion, and the evidence was considered inconclusive.^52^ Although evidence is inconclusive, it is recommended to prepare an adherence plan with the individual and discuss this at each healthcare encounter (ideally at monthly follow-ups), especially for children on TPT. The WHO guidelines for the treatment of drug-susceptible TB disease should be taken as a model, using interventions to support adherence, including mobile phone-based support systems and personalised approaches, because they could also be applied to TPT.

#### Evaluation at end-of-treatment and beyond

The following treatment outcomes should be reported at the end-of-treatment:

- Treatment completion – the recommended number of doses that has been successfully administered within the required duration of treatment according to the selected TPT regimen;
- Failed – development of TB disease any time while on TPT;
- Died – death for any reason while on TPT;
- Lost to follow-up – TPT interrupted by the person for ≥8 consecutive weeks for 6H, ≥4 consecutive weeks for 3HP, 3HR and 4R, and 10 consecutive days for 1HP;
- TPT discontinuation due to toxicity – by clinician due to adverse effects or drug–drug interactions, with or without restart or switching of regimen;
- Not evaluated – such as records lost, transfer to another health facility with no record of TPT completion.

On completion of treatment, all persons (including those in HIV programmes) should receive a certificate for completion of a TPT regimen. In low-incidence countries, the certificate would prevent people from unnecessary retreatment when attending unlinked medical services. This is particularly relevant for low-incidence countries where reinfections are uncommon. An electronic application for mobile phones has been created by the WHO to guide national programmes on critical data to collect along the TBI care pathway as an accessory to monitoring and evaluation.^53^ The feasibility of measuring efficacy of preventive therapy should be assessed. This would require the collection of information about the occurrence of TB disease in people who have received TPT. Measurement of efficacy also requires patients registered for TB treatment to be asked about their history of starting or completing TPT, and updating TBI registers with pre-existing data or the cross-linking of registers (e.g., TBI registers compared with TB treatment or mortality registers). For people who develop TB disease after, or well into a TPT course, drug susceptibility testing is critically important. National surveillance systems for anti-TB drug resistance to drugs used for TPT may need to be strengthened in countries scaling up programmatic management of TB preventive therapy (PMTPT).

## STANDARD 8

### A TBI screening and testing register should be kept to inform the cascade of care

**Figure 2:**
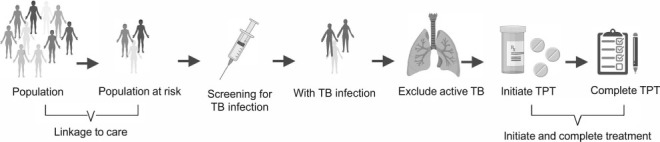
Cascade of care for TB prevention. TPT = TB preventive therapy. Created using Biorender (BioRender.com).

TBI testing and treatment is recommended by the WHO as a core intervention for TB elimination.^13^,^14^,^20^,^54^ Monitoring and evaluation of activities for TPT and for TB screening should be aligned to promote synergies and limit duplication. In addition, barriers to healthcare, especially for migrants where health facilities may not be accessed, should be removed.^55^ The implementation of a TBI register, including specific key variables, is essential to describe, measure and evaluate the cascade of care (Figure 2). Programmatic implementation and scale-up of TPT requires strengthening of each step in the cascade of care, starting from the identification of the target population to the completion of TPT. The TBI register represents a best-practice standard for patient follow-up, and programmatic planning; it is also an audit trail for clinical governance to evaluate programmatic effectiveness of the intervention.^20^,^56^,^57^ The register will ensure that individual patients receive appropriate follow-up; clinical management of patients is also useful for programmatic reasons to avoid duplication of notifications, inform the public health response and determine the direction of the programme (including change of target groups or specific support interventions to improve uptake or completion).^58^ Individual data are preferred over aggregated data, but this depends on local arrangements.

**Figure 3:**
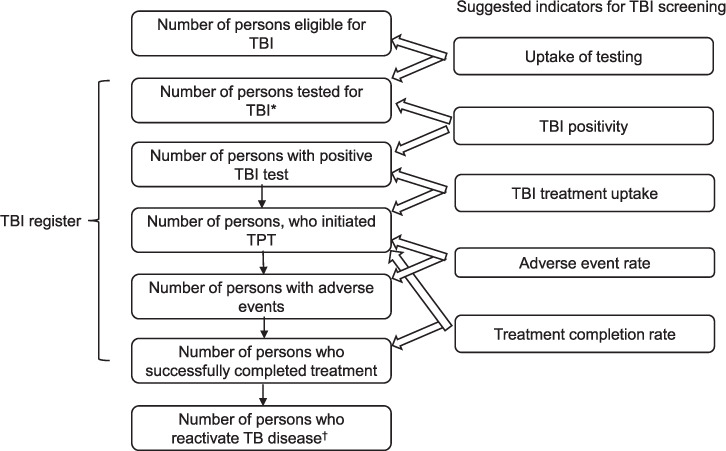
Indicators for TBI screening and elements of the TBI register. Children under 5 years of age and PLHA may not have had a test before TBI treatment initiation. The TBI treatment uptake here would be number of persons who initiated TPTover persons eligible for TBI in this age group. *Patients eligible for TBI testing can opt-out of the TBI register; ^†^Patients who reactivate TB disease after or during TBI treatment should be linked to the national TB surveillance. TBI = TB infection; TPT = TB preventive therapy; PLHA = patients living HIV/AIDS.

Elements of the TBI register is shown in Figure 3. Data protection laws and other restrictions at the country or regional level may limit the type of data that can be collected and may necessitate amending modalities of data collection. Here, we outline the components of such a TBI register. Ideally, the individual’s consent is taken at the beginning of the screening process, with opt-out consent the preferred option. Outcomes should be carefully recorded, including adverse effects and the occurrence of TB disease. Systems should be in place to ensure TB status can be updated in the register, even after follow-up or successful completion of TBI therapy. Effective implementation of the TBI register allows continuous monitoring of activities and can help programme managers to assess the performance of the TPT components.

The WHO recommends three main indicators for global evaluation of TPT activities (Table 7):^20^

**Table 7:** Description of key monitoring indicators for systematic screening and TPT

Indicator	Definition	Numerator	Denominator
Contact investigation coverage (WHO global and regional indicator): contact investigation identifies people recently exposed to TB with a high risk of developing TB disease; this is one of the top 10 indicators of the WHO End TB Strategy	Number of contacts of bacteriologically confirmed TB patients evaluated for TB disease and TB infection out of those eligible, expressed as a percentage	Total number of contacts of bacteriologically confirmed TB patients who completed evaluation for TB disease and TB infection during the reporting period	Total number of contacts of bacteriologically confirmed TB patients during the reporting period
TPT coverage (WHO and UNHLM indicator): this indicator should include all people deemed at risk and eligible for TPT; disaggregation by PLHA (newly or currently enrolled on ART), and by close contacts aged <5 years and ≥5 years allows reporting to WHO for monitoring of UNHLM targets; disaggregation by TPT regimen helps assess the uptake of shorter rifamycin-containing regimens and inform the procurement and supply chain management	Number of individuals initiated on TPT out of those eligible, expressed as a percentage	Total number of individuals eligible for TPT who initiated treatment during the reporting period	Total number of individuals eligible for TPT during the reporting period
TPT completion: this indicator helps assess the quality of PMTPT implementation, as the effectiveness of TPT depends upon its completion. When reported alongside the other two indicators above, the reporting period should be earlier (e.g., 6 months or 12 months preceding) to allow time for completion of the TPT	Number of individuals completing TPT out of those initiating treatment, expressed as a percentage	Total number of individuals who completed a course of TPT* during the reporting period	Total number of individuals who initiated a course of TPT during the reporting period

TPT =TB preventive therapy; ART=antiretroviral therapy; UNHLM=United Nations General Assembly High-Level Meeting on Ending TB; PLHA=people living with HIV/AIDS; PMTPT = programmatic management of TB preventive therapy.

1. Contact investigation coverage: percentage of contacts of bacteriologically confirmed TB patients who were evaluated for TB disease and TBI out of those eligible;
2. TPT coverage: percentage of individuals that initiated on TPT out of those eligible;
3. TPT completion: percentage of individuals completing TPT out of those initiating treatment.

An example of a recording and reporting form is presented in Supplementary Table S1.

## RESEARCH PRIORITIES

Significant improvement to the current diagnostic and treatment programmes will be necessary for effective widespread implementation. Areas where research gaps have been clearly identified by the scientific community are listed below.^59^

### Target populations

People included in target populations for PMTPT recommended by the WHO only represent a small fraction of the estimated one quarter of the world’s population with TBI. Expanding testing and treatment activities to additional target populations will be essential to increase the impact of the intervention and strengthen its contribution to the TB elimination strategy. Quality evidence from clinical trials is lacking for indigenous populations, people with DM, those with harmful use of alcohol, tobacco smokers, the underweight, and those exposed to silica, on steroid treatment, with rheumatological diseases or with cancer. Both direct measurement of the incidence of TB disease and methods for measuring the risk for TB disease should be explored, such as the evaluation of human RNA transcripts (correlate of risk) to investigate the risk of TB reactivation.^60^–^62^

### Improved diagnostic tests and performance of TBI tests in at-risk populations

Diagnostic tests with improved performance and predictive value for progression to TB disease are critically needed. The performance of TBI tests should be evaluated in various risk groups to understand how available tools should be optimally used in each population (e.g., combined or sequential use of TST and IGRA). Diagnostic tests for reinfections in treated patients would be highly desirable in high-transmission settings.

### Ruling out TB disease

This step of the cascade is particularly weak in resource-constrained settings. Operational and clinical studies should be conducted on new reliable and affordable techniques that can reduce health system and patient barriers (e.g., use of mobile chest radiography; computer-assisted radiography reading; C-reactive protein levels).^63^ Reliable human RNA transcripts (correlate of risk) that can predict progression to TB disease are urgently needed.

### Treatment options for TBI

Research to find shorter, better-tolerated TPT regimens than those currently recommended remains a priority, including treatment options after exposure to drug-resistant TB patients as given in Table 8. More evidence on the ultra-short 1-month regimen of RPT and INH is required, particularly in HIV-negative adults, injection drug users on methadone therapy, children aged <2 years and in pregnant women. A direct comparison of 1HP vs. 3HP for safety, effectiveness and cost-effectiveness will be useful. Pharmacokinetics studies to establish interactions with newer HIV drugs are required. In addition, the durability of protection of different TPT regimens, including those containing long-acting injectables, need to be evaluated in settings in which TB is highly incident, including the efficacy of repeated courses of TPT. Studies on the preference of different stakeholders for different regimen characteristics would be helpful.

**Table 8:** The global clinical development pipeline for new drugs and regimens to treat TB infection, August 2021 (reproduced with permission from the WHO^1^).

Phase I/II	Phase III/IV
DOLPHIN and DOLPHIN TOO NCT03435146	PHOENIx NCT03568383
IMPAACT P2001 NCT02651259	TB-CHAMP ISRCTN92634082
TBTC Study 35 NCT03730181	ASTERoid, Phase II/III NCT03474029
Higher-dose rifampicin for 2 months vs standard dose rifampicin-2R2 NCT03988933	SDR: 1HP vs. 3HP NCT04094012
Impact of 3HP on pharmacokinetics of tenofovir alafenamide-YODA NCT03510468	V-QUIN trial ACTRN12616000215426
Impact of 3HP on pharmacokinetics of dolutegravir and darunavir, with cobicistat NCT02771249	WHIP3TB NCT02980016
Drug-drug interactions between rifapentine and dolutegravir in HIV/LTBI co-infected individuals NCT04272242	1HP vs. 3HP among people living with HIV NCT03785106
	1HP vs. 3HP among people not infected with HIV NCT04703075

IMPAACT = International Maternal Pediatric Adolescent AIDS Clinical; HP = isoniazid+rifapentine; LTBI = latent TB infection.

### Monitoring adherence, adverse events and treatment completion

Carefully designed studies, including RCTs, are required to generate evidence on the effectiveness and quality-of-life impact of context-specific interventions to enhance adherence and completion of treatment in specific risk groups. Integration of PMTPT into other health care services (i.e. for PLHA, people with DM, people on immunosuppressive therapy) should be explored. Prospective RCTs are required to determine the incremental benefits of routine monitoring of liver enzyme levels over education and clinical observation alone for preventing severe clinical adverse events, with stratification of the evidence by at-risk population. Use of digital technologies to improve adherence should be carefully explored, including the use of mobile texting for asymptomatic individuals. The same technologies should be applied to treatment completion monitoring.

### Cost-effectiveness

Although there are some studies comparing 3HP vs. 6H, a comprehensive appraisal of the cost-effectiveness of TBI management stratified by population group, and type of regimen or intervention is warranted. Cost-effectiveness analysis using parameters from different resource settings could allow better planning for the extension of a PMTPT strategy at a national or local level.

### Contacts of people with MDR-TB

The current WHO recommendation on MDR-TB preventive treatment responds to an urgent need from the countries but is based on very weak evidence. The results of currently ongoing RCTs, including those listed in Table 8, will need to be translated into a policy update as soon as possible. The need, effectiveness and safety of TPT for contacts of people with MDR-TB, pre-XDR-TB and XDR-TB should be evaluated under operational conditions.

### Programme management

Implementation research on context-specific barriers and facilitators is needed for different TBI regimens to explore dimensions such as acceptability, feasibility, equity and resource use. Research is also needed on service delivery models to improve management. Household implementation models could increase the effectiveness and efficiency of delivery of interventions.^64^,^65^ Future trial evidence could guide how to optimise contact-tracing strategies in households and other congregated settings where transmission may occur, such as the workplace, schools and penitentiary institutions.
